# Hepatic Lipid Accumulation and Dysregulation Associate with Enhanced Reactive Oxygen Species and Pro-Inflammatory Cytokine in Low-Birth-Weight Goats

**DOI:** 10.3390/ani12060766

**Published:** 2022-03-18

**Authors:** Tingting Liu, Rui Li, Nanjian Luo, Peng Lou, Sean W. Limesand, You Yang, Yongju Zhao, Xiaochuan Chen

**Affiliations:** 1 Chongqing Key Laboratory of Herbivore Science, College of Animal Science and Technology, Southwest University, Chongqing 400715, China; liu_19971021@163.com (T.L.); lirui_214@163.com (R.L.); loupeng@stu.scu.edu.cn (P.L.); youygz@163.com (Y.Y.); zyongju@163.com (Y.Z.); 2 College of Basic Medicine, Zunyi Medical University, Zunyi 563000, China; luonanjian28@sina.cn; 3 School of Animal and Comparative Biomedical Sciences, The University of Arizona, Tucson, AZ 85721, USA; limesand@arizona.edu

**Keywords:** low birth weight, goat, liver, lipid accumulation, antioxidant capacity

## Abstract

**Simple Summary:**

In livestock, a low birth weight (LBW) has a broad impact on neonatal survival, growth performance, and metabolic health in adult life. The liver plays an important role to regulate lipid metabolism, but the development of hepatic dyslipidemia associated with LBW is still unknown in goats. Herein, we evaluated lipid and metabolic status of LBW livers in contrast to those of newborns with normal birth weight. RNA sequencing was used to screen potential dysregulated functional genes involved in hepatosteatosis. Results showed lower antioxidant capacity, enhanced pro-inflammatory cytokine, and increased hepatic lipid accumulation in LBW goats associated with impaired regulatory machineries. Understanding the knowledge of intrinsic mechanism underlying hepatic dyslipidemia in LBW goats could provide important implications for promoting efficiency of production and health in their later life.

**Abstract:**

Occurrence of low birth weight (LBW) is a major concern in livestock production, resulting in poor postnatal growth, lowered efficiency of feed utilization, and impaired metabolic health in adult life. In the southwest region of China, birth weight of indigenous strains of goats varies seasonally with lower weights in summer and winter, but the metabolic regulation of the LBW offspring is still unknown. In this study, by comparing LBW goats to normal birth weight group, we examined hepatic lipid content in association with regulatory mechanisms. Histological studies showed higher microvesicular morphology in the liver of LBW goats in accompany with a significantly higher level of hepatic free fatty acids, total triglycerides, and cholesterols. Lipid metabolism impairment, increased oxidative stress, and inflammation were observed by transcriptome analysis. Meanwhile, Kyoto Encyclopedia of Genes and Genomes (KEGG) annotation further demonstrated lipid peroxidation, antioxidant pathway, and pro-inflammatory response involved in the hepatic lipid dysregulation from LBW group. Therefore, dysregulations of hepatic lipid metabolism, including fatty acid biosynthesis and degradation, lipid transportation, and oxidative stress, played important roles to contribute the lipid accumulation in LBW goats. Moreover, due to impaired antioxidant capacity, the oxidative damage could interact with persisting pro-inflammatory responses, leading to a higher risk of liver injury and metabolic syndromes in their adult life.

## 1. Introduction

Adverse environmental conditions, suboptimal fetal growth, and undernutrition may harm livestock, resulting in the occurrence of low birth weight (LBW) in animals [1]. Various LBW animal studies of rats, pigs, and lambs have indicated that LBW progeny have a greater risk of developing metabolic complications in their adult life, including impaired insulin secretion, glucose intolerant, insulin resistance, and dyslipidemia [2,3,4,5]. In the southwest China, the birth weight of indigenous goats (*Capra hircus*) during summer and winter is approximately 20% lower than annual average [6]. The pre-weaning mortality of those LBW newborns is also significantly higher than their normal birth weight (NBW) counterpart [6]. In goats, however, the impact of the LBW on postnatal metabolic and homeostatic status is still not clear.

The liver is an important metabolic organ that controls lipid, glucose, and energy metabolism. In rat pups with LBW, male newborns exhibit higher triglyceride and fatty acid synthase in liver [7]. They also have greater adiposity and suffer from metabolic syndromes in adult life [8]. LBW piglets show increased hepatic lipid accumulation associated with decreased lipase activity in liver [9]. Oxidative stress is regarded as one of the pathological mechanisms that causes various liver diseases. Recent studies found that complications in LBW animals were associated with reduced mitochondrial function and antioxidant response elements, leading to oxidative damage and inflammation in liver [10,11]. Activated by excessive reactive oxygen species (ROS), oxidative stress not only affects liver fatty acids oxidation and synthesis [12], but also stimulates the hepatic inflammatory pathway to promote cytokine secretion [13]. Therefore, we hypothesize that LBW goat kids have greater hepatic lipid accumulation associated with impaired hepatic lipid regulation and excessive ROS production. 

In the current study, we evaluated liver lipid profiles by comparing between the LBW and NWB newborn goats. Then, we investigated the intrinsic pathway associated with impaired lipid metabolism to understand the underlying regulatory mechanism. At one month of age, LBW kids exhibited higher hepatic total triglycerides (TG) and free fatty acids (FFA) levels that are associated with impairment of fatty acids biosynthesis and degradation, lipid transportation, and oxidative regulation. Furthermore, an observation of lower hepatic antioxidant capacity, as well as higher ROS and pro-inflammatory cytokine levels could lead to a higher risk of liver damage in their adult life. 

## 2. Materials and Methods

### 2.1. Animal Husbandry

All experiments were performed according to the principles and guidelines of the Southwest University Institutional Animal Care and Use Committee (2019, No. GB14925-2010). A Chinese indigenous goat breed (Dazu black goat) was used in our current study. Each twin was delivered from first parity ewe, and purchased from Dazu Ruifeng goat farm (Dazu, Chongqing, China). In total, 12 goats, half male and half female, were equally assigned to control and LBW groups. The criteria of selection NBW as control and LBW group is based on historic data from the farm [6]. All kids were transported to the laboratory in Southwest University (Beibei, Chongqing, China) at around 4–7 days of age. Then, they were fed with milk replacer (BaiNianLongTeng, Yunnan, China) until approximately 30 days of age. Goat kids were euthanized at 28~30 days of age by overdosing venous sodium pentobarbital (86 mg/kg). Organs and tissues were weighted, frozen in liquid nitrogen, and stored at −80 °C for further use.

### 2.2. Biochemical Parameters Assays

Hepatic malondialdehyde (MDA assay kit, A003-1-2), glutathione peroxidase (GPx, GSH-PX assay kit, A005-1-2), total triglycerides (Triglyceride assay kit, A110-1-1), total cholesterol (TC, total cholesterol assay kit, A111-1-1), and ATP content (ATP assay kit, A095-1-1) were determined by the colorimetric method according to the manufacturer’s instructions of Nanjing Jiancheng Bioengineering Institute (Jiangsu, China) [11]. Liver free fatty acids (YX-C-B400, SINOBESTBIO, Shanghai, China) and glycogen (BC0345, Solarbio, Beijing, China) were quantified by respective commercial kits, and measured by xMark™ Microplate Absorbance Spectrophotometer (Bio-Rad, Hercules, CA, USA) [14]. Protein concentrations of liver were measured by the Enhanced BCA Protein Assay Kit (P0010S, Beyotime, Shanghai, China). 

### 2.3. RNA Sequencing and Analysis

Liver samples (control, *n* = 3; LBW, *n* = 3) were randomly selected from each group, and submitted to the Biomarker Technologies (Beijing, China) for high throughput RNA sequencing (RNAseq). Generally, after isolating RNA from liver tissue, RNA integrity was assessed using the RNA Nano 6000 Assay Kit with the Agilent Bioanalyzer 2100 system (Agilent Technologies, Santa Clara, CA, USA). Sequencing libraries were generated by using NEBNext^®^ Ultra™ RNA Library Prep Kit (#E7770, New England Biolabs, Ipswich, MA, USA) following the manufacturer’s recommendations. Index codes were added to attribute sequences to each sample, and performed on a cBot Cluster Generation System using TruSeq PE Cluster Kit v4-cBot-HS (Illumia, San Diego, CA, USA) according to the manufacturer’s instructions. After cluster generation, the library preparations were sequenced on an Illumina platform (NovaSeq 6000, San Diego, CA, USA), and paired-end reads were generated.

The adaptor sequences and low-quality sequence reads were removed from the data sets. Raw sequences were transformed into clean reads after data processing. These clean reads were then mapped to the reference genome sequence (ARS1, GenBank assembly accession: GCA_001704415.1) by HISAT2 [15,16]. Only reads with a perfect match or one mismatch were further analyzed and annotated based on the reference genome. Genes were normalized according to StringTie [17], and gene expression was presented as fragments per kilobase of transcript per million mapped reads (FPKM), which was calculated by the following formula [18]:

$$FPKM=\frac{cDNA\;Fragments}{Mapped\;Fragments\;\left(Millions\right)\times Transcript\;Length\;\left(kb\right)}$$

Differential expression analysis of two samples was performed using the edgeR [19]. The false discovery rate (FDR) < 0.05 & |log_2_(fold change)| ≥ 1.0 was set as the threshold for significantly differential expression [20]. Gene function was annotated based on the Gene Ontology (GO) database (Accessed date: 24 February 2020, http://www.geneontology.org/). GO enrichment analysis of the differentially expressed genes (DEGs) was implemented by the GOseq R packages based Wallenius’ non-central hyper-geometric distribution [21]. GO and Kyoto Encyclopedia of Genes and Genomes (KEGG) terms with corrected *p* < 0.05 were defined as significantly enriched by commonly expressed genes (CEGs) and differently expressed genes (DEGs) [22].

### 2.4. Quantitative Analysis of mRNA Expression and Mitochondrial DNA

Relative expression levels of the DEGs from RNAseq were evaluated by real-time qPCR in liver from groups. Generally, total RNA was extracted from liver by TRIzol Reagent (Thermo Fisher Scientific, Waltham, MA, USA). The concentrations of RNA were determined by using NanoDrop™ One spectrophotometer (Thermo Fisher Scientific, Waltham, MA, USA). Then, mRNA was reverse transcribed into cDNA by following to the manufacture’s steps of PrimeScript™ RT reagent Kit with gDNA Eraser (RR047A, TaKaRa, Beijing, China). Primer sequences are presented in Table S1. The relative expression of mRNA was determined by using TB Green^®^ Premix Ex Taq™ II (RR820A, TaKaRa) with the CFX96 Touch™ Real-Time PCR Detection System (Bio-Rad). The qPCR thermal cycling conditions were 95 °C for 30 s, then 40 cycles of 95 °C for 5 s, 60 °C for 30 s, and 72 °C for 30 s. Relative mRNA expression levels were determined from the threshold cycle (Ct) values using the 2^−ΔΔCt^ comparative method [23].

In the liver samples, total DNA was isolated by using SteadyPure Universal Genomic DNA Extraction Kit (AG21009, Accurate Biotechnology, Changsha, China). In order to quantify the amount of mitochondrial DNA (mtDNA) present per nuclear genome, we designed the primers from *Capra hircus* mitochondrial cytochrome B gene for mtDNA, and the primers from chromosome 11 for nuclear DNA (nDNA) (Table S1) [24,25]. The mtDNA/nDNA ratio evaluated by qPCR was used to analyze mitochondrial density [26].

### 2.5. Western Blot Analysis

Frozen liver tissue was homogenized in RIPA Buffer (CW2333S, CWBIO, Jiangsu, China) with protease inhibitor (CW2200, CWBIO). After they were separated by SDS-PAGE (P0012, Beyotime) and transferred on PVDF membranes (IPVH00010, Immobilon-P, Merck Millipore, Burlington, MA, USA), liver proteins were incubated with primary antibodies of tumor necrosis factor alpha (TNFα, 33207M, 1:1000, Boiss, Beijing, China), nuclear factor erythroid 2-related factor 2 (Nrf2, 16396-a-AP, 1:1500, Proteintech, Hubei, China), OxPhos (45-8099, 1:1000, Thermo Fisher Scientific), and β-actin (BS-0061R, 1:1000, Boiss) overnight. Then, the membranes were incubated with either the goat anti-mouse IgG (H + L) (A0216, 1:5000, Beyotime) or the goat anti-rabbit IgG (H + L) (A0208, 1:5000, Beyotime) for 2 h. Bands were visualized by Immobilon Western HRP Substrate (WBKLS0500, Millipore), obtained by ChemiDoc™ XRS+ Imaging System (Bio-Rad), and analyzed by ImageJ software (Version 1.53n, National Institutes of Health, Bethesda, MD, USA). 

### 2.6. Histological Analyses

Liver tissues were fixed in 4% paraformaldehyde for 48 h at room temperature. After being consecutively dehydrated in 70%, 90%, and 100% ethanol, liver tissues were embedded in paraffin and cut into slices measuring 5 μm in thickness. The slices were stained by hematoxylin-eosin (HE), according to the manufacturer’s protocol (G1005, Servicebio, Hubei, China). Histological analysis of steatosis was visualized with the microscope (DP74, Olympus, Tokyo, Japan), digitally captured, and analyzed with cellSens software (Version 3.1.1, https://www.olympus-lifescience.com/en/software/cellsens/#!cms[focus]=cmsContent6017, accessed on 1 February 2022 Olympus).

### 2.7. Statistical Analysis

All of the data between groups were compared by using paired Student’s *t*-test. Pearson’s correlation coefficient was used to evaluate the determination of genes expression between RNAseq and qPCR by Prism 8.0.2 (GraphPad Software, San Diego, CA, USA). Statistical analysis was processed by SPSS Statistics 19.0 (SPSS Inc., Armonk, NY, USA). Values are given as mean ± SEM, and *p* < 0.05 was considered significant.

## 3. Results

### 3.1. Weights

The average birth weight of LBW neonatal goats (1.64 ± 0.11 kg) was significantly less than the NBW group (2.50 ± 0.16 kg, *p* < 0.01). After necropsy, LBW bodyweights were 23% lower than control group, and the carcass weights of LBW goats were still 25% lower than the control group (*p* < 0.05 Table 1). The average daily gain of body weight was not different between the LBW goats compared to the control (data not shown). 

### 3.2. Morphological and Metabolic Features in Liver

**Figure 1:**
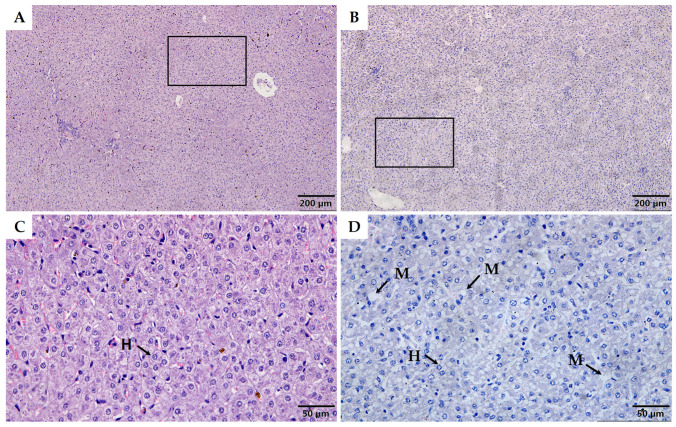
Histological analysis of the liver tissue in control (**A**,**C**) and LBW (**B**,**D**) goat kids. H with arrowheads indicate hepatocyte; M with arrowheads indicate microvesicular steatosis.

**Figure 2:**
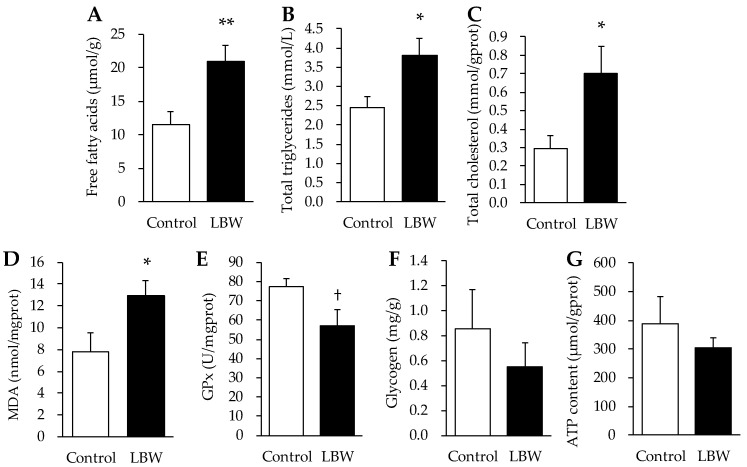
Metabolic features of free fatty acids (**A**), total triglycerides (**B**), total cholesterol (**C**), MDA (**D**), GPx (**E**), glycogen (**F**), and ATP content (**G**) were determined in liver from control and LBW kids. **, *p* < 0.01; *, *p* < 0.05; †, *p* = 0.057. MDA, malondialdehyde; GPx, glutathione peroxidase; LBW, low birth weight.

Histological analysis of HE-stained liver sections showed that LBW kids presented small intracellular fat vacuoles and liposomes, indicating mild to moderate microvesicular steatosis in liver (Figure 1). Further, the level of FFA, TG, and TC were significantly higher in livers from LBW kids compared to control kids (Figure 2A–C). LBW kids exhibited 1.67-fold higher concentrations of MDA (*p* < 0.05, Figure 2D) than control kids. A trend of lower GPx was observed in LBW kids (*p* = 0.057, Figure 2E). Hepatic glycogen and ATP content were not different between the groups (Figure 2F,G). 

### 3.3. Differential Gene Expression of RNAseq

**Figure 3:**
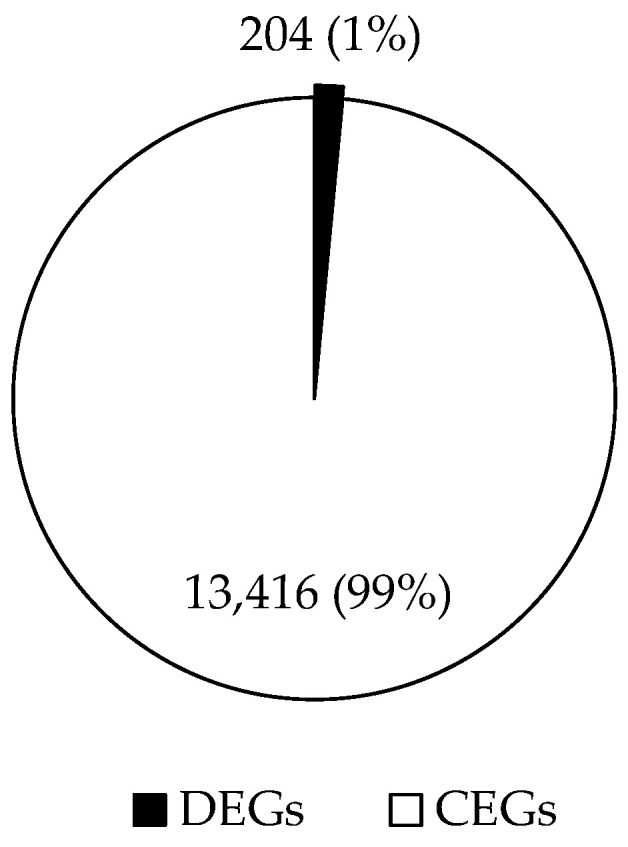
The distribution of commonly expressed genes (CEGs) and differently expressed genes (DEGs). There were 13,620 annotated transcripts identified in liver from the control and LBW kids, with 204 DEGs and 13,416 CEGs.

**Figure 4:**
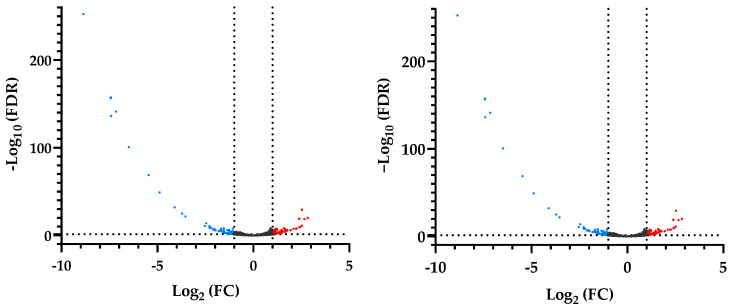
Volcano plot of global genes expression. The statistically significant genes with ≥ 1.5-fold change and false discovery rate of less than 0.05 are plotted in red (up-regulated genes) and blue (down-regulated genes). FDR, false discovery rate; FC, fold change.

**Figure 5:**
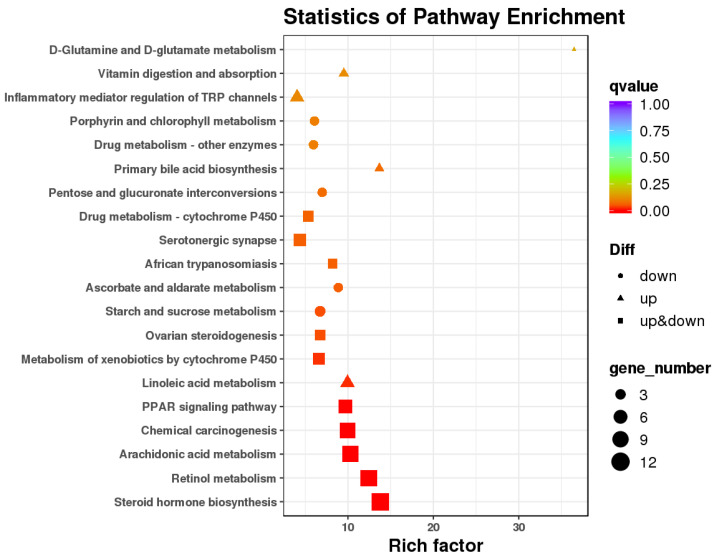
KEGG pathway enrichment analysis of DEGs.

RNAseq reads mapped well to the reference goat genome, with 97.01–97.97% aligning concordantly. There were 13,620 annotated transcripts identified in livers from the control and LBW kids (Figure 3). There were 204 DEGs with 86 genes up-regulated and 118 down-regulated in LBW livers, compared to the control group (Figure 4). KEGG annotated pathways related to hepatosteatosis were involved in lipid metabolism, oxidative regulation, and inflammatory signaling (Figure 5, Table 2). In total, 32 differential unknown genes with FASTA format are listed in Supplemental Table S2. Statistics of sequencing data output, including quality score and size of trimmed sequence, is presented in Table S3.

### 3.4. Gene Expressions in Liver

**Figure 6:**
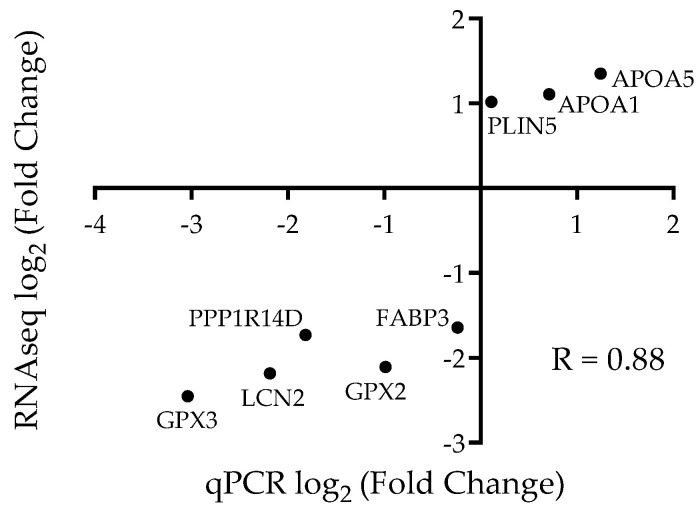
Gene expression determined by RNAseq reflects qPCR. The log_2_ transformed fold changes are plotted for RNAseq results (abscissa) and qPCR results (ordinate). Fold change of eight genes determined by qPCR correlated significantly (*p* < 0.0043) with the fold change determined by RNAseq. The slope of best fit after Pearson correlation was 0.88, with a 95% confidence interval of 0.45 to 0.98.

Relative expression levels of eight genes from 204 DEGs was evaluated through real-time qPCR in an expanded cohort of LBW and control livers, and compared with the RNAseq results. The fold changes for these two methods correlated positively with R value = 0.88 (*p* < 0.01, Figure 6), indicating the differential genes from RNAseq exhibited the accordant expression by qPCR evaluation. 

**Figure 7:**
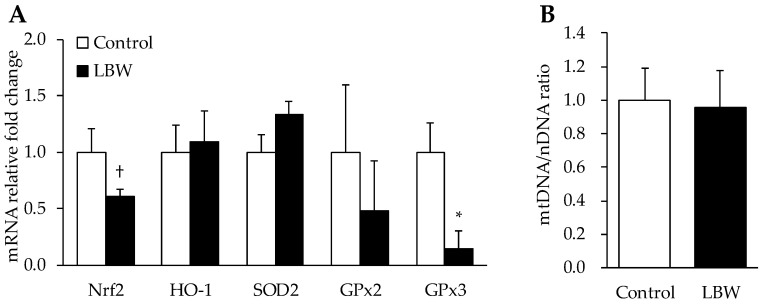
mRNA expression levels in livers of control and LBW kids. (**A**), relative fold changes for nuclear factor erythroid 2-related factor 2 (Nrf2), heme oxygenase 1 (HO-1), superoxide dismutase 2 (SOD2), and glutathione peroxidase (GPx). *, *p* < 0.05; †, *p* = 0.064. (**B**), quantifications of mtDNA/nDNA.

Relative mRNA expression of GPx3 was 86% lower in LBW kids (*p* < 0.05), but a trend of lower Nrf2 (*p* = 0.064) mRNA expression was observed in LBW kids compared to the control group. No differences in mRNA expression were observed for heme oxygenase 1 (HO-1), superoxide dismutase 2 (SOD2), or GPx2 (Figure 7A). The ratio of mtDNA to nDNA was also determined by qPCR. The ratio of mtDNA/nDNA was not different between the two groups (Figure 7B).

### 3.5. Hepatic Protein Expressions

**Figure 8:**
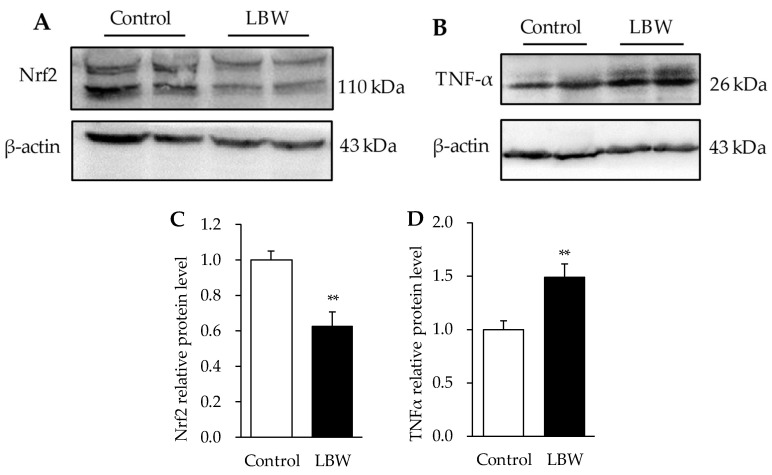
Relative protein expression of Nrf2 and TNFα in liver. (**A**,**B**), representative immunoblots for Nrf2 and TNFα between control and LBW kids. Original Western Blot could be found as Figures S1–S4. (**C**,**D**), relative protein concentration of Nrf2 and TNFα normalized by abundance of β-actin. Nrf2: nuclear factor erythroid 2-related factor 2; TNFα: tumor necrosis factor alpha. **, *p* < 0.01. Original Western Blot figures can be found at Supplementary Material.

**Figure 9:**
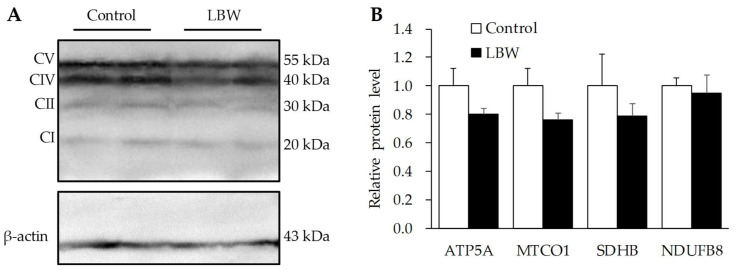
Relative protein expression level of oxidative phosphorylation complexes in livers. (**A**) representative immunoblots for the complexes I (CI, NDUFB8), II (CII, SDHB), IV (CIV, MTCO1) and V (CV, ATP5A) in livers of control and LBW kids. (**B**) relative protein concentration of oxidative phosphorylation complexes normalized by abundance of β-actin. Original Western Blot could be found as Figures S5 and S6.

The LBW kids exhibited relatively 63% lower protein abundance of Nrf2, and 1.49-fold higher of TNFα in the liver, compared to the control kids (*p* < 0.01, Figure 8). However, the protein expressions of oxidative phosphorylation complexes in mitochondria, (CI-NDUFB8), (CII-SDHB), (CIV-MTCO1), and (CV-ATP5A) were not different between the two groups (Figure 9).

## 4. Discussion

In the present study, we found that LBW goat kids exhibited preliminary signs of lipid accumulation in liver, including enhanced concentrations of hepatic FFA and TG, as well as the occurrence of vacuole lipid droplets in liver tissue. The key regulatory signaling pathways related to hepatic lipid metabolism, oxidative regulation, and inflammation were impaired in LBW kids compared to NBW kids. The lower antioxidant capacity was expected to play a major role to contribute the excessive ROS induced by lipid peroxidation in the liver. Together with the persistently increased pro-inflammatory cytokine, these metabolic complications could further lead to liver injury in their later life.

### 4.1. Hepatic Lipid Accumulation and Oxidative Stress in LBW Goat Kids

In clinical studies, LBW is defined as birthweight less than the 10th percentile at gestational delivery [1,27]. Epidemiological studies have shown that LBW is associated with a greater risk for development of non-alcoholic fatty liver disease in both children and adults [28,29]. Domestic animal studies also have shown that LBW newborns have organ dysfunction and abnormal development in the liver [1]. In our current study, the average weight of LBW goats is 23% lower than the NBW control at necropsy (Table 1), which is consistent with previous findings for LBW goats and lambs [6,30]. By comparing the necropsy weight to birth weight, we found that the growth efficiency was 86.8% for NBW and 118.9% for LBW, respectively. Even though the average daily gain was not different between the two groups, we still cannot rule out the possibility of rapid growth in LBW group. Postnatal accelerated growth, also called catch-up growth, occurs frequently in LBW human and animals. The detrimental effects of catch-up growth have been associated with dyslipidemia, obesity, and glucose intolerance [1,31]. Thus, further studies are necessary to better interpret the hepatic lipid dysregulation caused by either prenatal programming or postnatal rapid growth. Currently, our study was more focused on evaluating liver lipid profiles between NBW and LBW, and investigating the underlying mechanism.

In the liver tissue of LBW goats, we found histological signs of microvesicular steatosis (Figure 1) associated with significantly higher lipid profiles, including FFA and TG (Figure 2), indicating the existence of developing liposomes and lipid accumulation in liver. Similar to the results in guinea pigs and rats [32,33], the hepatic total cholesterol content was also increased in our LBW goat kids compared to the NBW group (Figure 2C). In order to fully understand the underlying regulatory mechanism, we explored liver transcriptome data associated with lipid metabolism, and discovered that multiple pathways, including fatty acid biosynthesis, adipocytokine signaling, and fatty acid degradation, were differentially affected in LBW liver (Table 2). These findings were similar to those in the study of LBW rats induced by bilateral uterine artery ligated, in which LBW offspring display higher expression of acetyl-CoA carboxylase, the rate-limiting enzyme of fatty acid synthesis, in the liver at 21 days of age [34]. Meanwhile, the LBW rats exhibited greater hepatic cholesterol accumulation with impaired regulation of lipid transport in their postnatal life [33]. Thus, our findings of hepatic lipid accumulation and dysregulation begin to reveal the development of fatty liver in LBW goats.

Besides the lipid metabolism, KEGG annotation further indicated dysregulation of oxidative regulation and inflammation in LBW livers. Oxidative stress is one of the main factors in the pathogenesis of metabolic diseases, including hepatic lipid accumulation and dysregulation. On the other hand, hepatic lipid accumulation occurs when fatty acid uptake and synthesis surpass oxidative capacity in liver [35]. As the marker of lipid peroxidation [36], hepatic MDA level was observed to be higher in LBW goat kids (Figure 2D), and there was a trend of lower GPx activity in LBW kids (Figure 2E). GPx is a potent enzyme with peroxidase activity able to reduce lipid hydroperoxides and protect the organism from oxidative damage [37]. Studies of LBW piglets show signs of oxidative damage that was induced by lower antioxidant capacity in the liver [38]. Moreover, these experiments demonstrate a close associate with hepatic dyslipidemia and dysregulated oxidative stress [39,40]. Moreover, small for gestational age newborns have increased MDA with lower antioxidants in comparison to the appropriate for gestational age babies [41]. Hence, our data provides evidence that greater lipid accumulation in LBW livers might result from persisting oxidative damage caused by higher ROS exposure concurrent with lower antioxidant capacity. 

### 4.2. Enhanced ROS Caused by Lower Antioxidant Capacity Damage Hepatic Lipid Regulation

Oxidative stress is mainly caused by an imbalance between the production of ROS and the corresponding antioxidant-induced protective mechanisms [42]. Nrf2 is a transcription factor that regulates cellular redox status. It is coupled with the antioxidant-response element to mediate stress-stimulated induction of antioxidant by activating GPx expression [37]. The treatments of antioxidative dietary supplementations, such as dihydroartemisinin and curcumin, upgrade the Nrf2 pathway and alleviate the ROS production and oxidative damage in LBW piglets [11,43]. In our LBW study, the mRNA expression of GPx3 was significantly lower in LBW goat kids than in the NBW group (Figure 7A). Moreover, there was a trend of decreasing Nrf2 mRNA expression (Figure 7A), and a significantly lower Nrf2 protein level in LBW livers (Figure 8A,C). Since LBW rats exhibit hepatic mitochondrial dysfunction and oxidative damage four weeks after birth [10], we further investigated the hepatic mitochondrial density and function to fully understand the imbalance of oxidative regulation in LBW goats. However, neither the mtDNA/nDNA ratio, the indicator of mitochondrial density, nor the expression of rate limited enzymes in mitochondrial respiration chain complex was changed (Figure 7B and Figure 9). Based on the above findings, this hepatic oxidative damage was mainly caused by down regulation of Nrf2-GPx pathway, resulting in a lower antioxidant capacity in LBW kids. 

In addition to influencing lipid metabolism directly, chronic ROS exposure could persistently activate pro-inflammatory cytokine generation in liver, and these cytokines, in turn, would stimulate higher ROS production and exacerbate hepatosteatosis [35]. The study of guinea pigs indicated that LBW offspring with a controlled diet have an increased inflammatory marker, TNFα, and exhibit minimal lobular inflammation, as well as portal fibrosis [44]. In LBW animals, improving hepatic antioxidant capacity and inflammation could alleviate hepatic lipid accumulation by postnatal diet supplement [40,45]. Hence, in our LBW goat kids, the higher hepatic TNFα level (Figure 8B,D) might be caused by the interaction between impaired redox signaling and innate activated inflammatory responses, resulting in further exacerbation of liver injury development.

## 5. Conclusions

**Figure 10:**
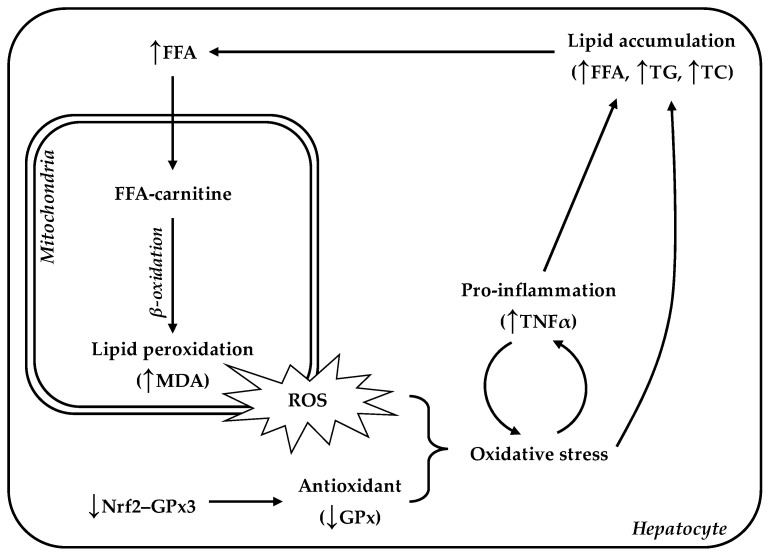
Hepatic lipid accumulation was associated with impairment of lipid metabolism and oxidative regulation in LBW goats. Overproduction of ROS and lower antioxidant capacity could contribute oxidative stress, which interacts with pro-inflammation cytokine, resulting in persistent lipid dysregulation and hepatosteatosis in the LBW goat’s adult life. FFA, free fatty acid; TC, total cholesterol; TNFα, tumor necrosis factor alpha; MDA, malondialdehyde.

LBW animals are at greater risk for developing oxidative stress-induced hepatosteatosis. The impairment of antioxidant capacity and excessive ROS exposure not only directly damage hepatic lipid regulation, but also trigger hepatic inflammatory responses, leading to irretrievable liver damage with lipid accumulation in the liver in later life (Figure 10). In these LBW animals, early intervention with an appropriate intake of antioxidant additives could relieve the level of oxidative stress, along with ameliorating the metabolic diseases [46,47].

## Figures and Tables

**Table 1:** Growth performance and organ weight of goat kids at one month of age.

Necropsy	Control	LBW
Body weight, kg	4.67 ± 0.44	3.59 ± 0.26 *
Carcass weight, kg	3.40 ± 0.34	2.57 ± 0.26 *
Brain, g	65.89 ± 2.93	56.62 ± 1.61 **
Heart, g	30.21 ± 1.85	24.88 ± 1.64 *
Liver, g	137.92 ± 10.68	108.77 ± 5.92 **
Lungs, g	81.83 ± 5.65	69.17 ± 3.83 *
Relative organ mass, g/kg		
Brain	14.42 ± 0.88	16.19 ± 1.32
Heart	6.55 ± 0.28	6.98 ± 0.31
Liver	29.95 ± 1.90	31.01 ± 2.48
Lungs	17.77 ± 0.98	19.62 ± 1.44

**, *p* < 0.01; *, *p* < 0.05. LBW, low birth weight.

**Table 2:** GO analysis and KEGG annotated pathways of DEGs associated with lipid metabolism, oxidative regulation, and inflammation.

Functional Description	KEGG Pathway	KEGG ID	Gene Category
Lipid metabolism	PPAR signaling pathway	KO03320	FABP3 (fatty acid-binding protein) PLIN5 (perilipin-5 isoform X1) LOC102173339 (7-alpha-diol 12-alpha-hydroxylase) APOA5 (apolipoprotein A-V) LOC102179867 (apolipoprotein A-I) APOA1 (apolipoprotein A-IV) LOC102187785 (cholesterol 7-alpha-monooxygenaseC)
	Fat digestion and absorption	KO04975	LOC102179867 (apolipoprotein A-I) APOA1 (apolipoprotein A-I)
	Glycerophospholipid metabolism	KO00564	LCAT (phosphatidylcholine-sterol acyltransferase precursor)
	Non-alcoholic fatty liver disease (NAFLD)	KO04932	Capra_hircus_newGene_23729 Capra_hircus_newGene_43596
	Fatty acid degradation	KO00071	LOC102181105 (alcohol dehydrogenase E chain isoform X1) LOC108633240 (cytochrome P450 4A11-like)
	FoxO signaling pathway	KO04068	LOC102172279 (serine protease HTRA3)
Oxidative regulation	Glutathione metabolism	KO00480	GPX2 (glutathione peroxidase 2) GPX3 (glutathione peroxidase 3)
	Metabolism of xenobiotics by cytochrome P450	KO00980	LOC102170823 (cytochromeP450 1A1) LOC102175204 (UDP-glucuronosyltransferase 2B4)
	Drug metabolism - cytochrome P450	KO00982	LOC102175204 (UDP-glucuronosyltransferase 2B4) LOC102181105 (alcohol dehydrogenase E chain isoform X1) LOC108635023 (UDP-glucuronosyltransferase 2B18-like)
	Oxidative phosphorylation	KO00190	Capra_hircus_newGene_23729 Capra_hircus_newGene_43596 Capra_hircus_newGene_43600 Capra_hircus_newGene_48268
Inflammation	Leukocyte transendothelial migration	KO04670	NCF4 (neutrophil cytosol factor 4 isoform X1) PTK2B (protein-tyrosine kinase 2-beta isoform X1)
	NF-kappa B signaling pathway	KO04064	LBP (lipopolysaccharide binding protein) Capra_hircus_newGene_7098
	Inflammatory mediator regulation of TRP channels	KO04750	LOC100861186 (cytochrome P450 2C31) OC102169851 (cytochrome P450 2C31) LOC106503891 (cytochrome P450 2C31) LOC108633308 (cytochrome P450 2C31-like)
	TNF signaling pathway	KO04668	LOC102184244 (interferon-inducible GTPase 1) 102188524 (leukemia inhibitory factor)

## Data Availability

The data presented in this study are available in the Supplementary Materials.

## Supplementary Materials

The following supporting information can be downloaded at: https://www.mdpi.com/article/10.3390/ani12060766/s1, Table S1: Nucleotide sequences of primers used for real-time qPCR. Table S2: List of differential expressed genes with unknown annotation. Table S3: Statistics of sequencing data output. Figures S1–S6: original Western Blot figures.
