# Supplementary material for: Hepatic Lipid Accumulation and Dysregulation Associate with Enhanced Reactive Oxygen Species and Pro-Inflammatory Cytokine in Low-Birth-Weight Goats
Source: Animals (Basel). 2022 Mar 18;12(6):766. doi: 10.3390/ani12060766 (PMC8944635; doi:10.3390/ani12060766)
Supplement: Supplementary file 1 [file animals-12-00766-s001.zip › Figure S - updated- WB.pdf]

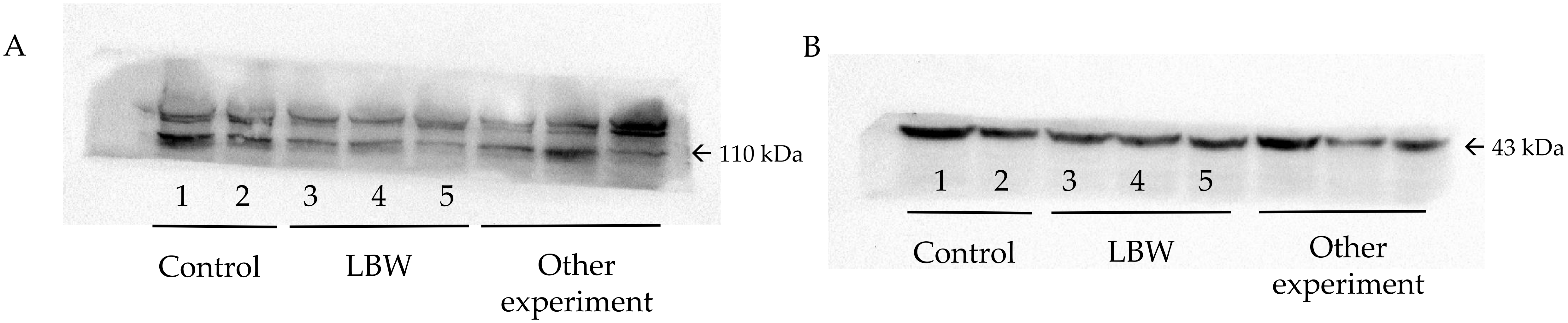

Figure S1. Immunoblot protein expression of Nrf2 (A, 110 kDa) and corresponding β-actin (B) in liver. Control, n = 2; LBW, n = 3. The density ratio of Nrf2 to β-actin are presented as follows, band#1, 1.08; band#2, 0.95; band#3, 0.74; band#4, 0.87; band#5, 0.60.

A

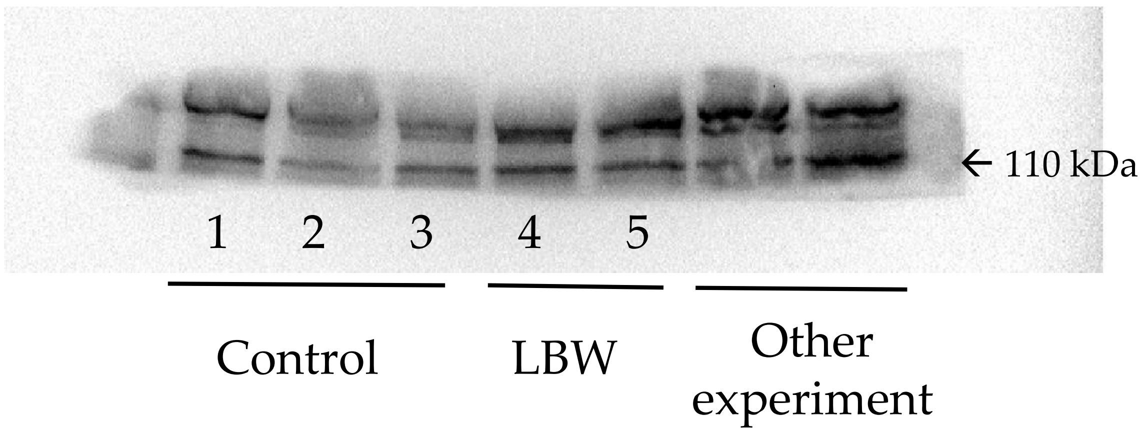

B

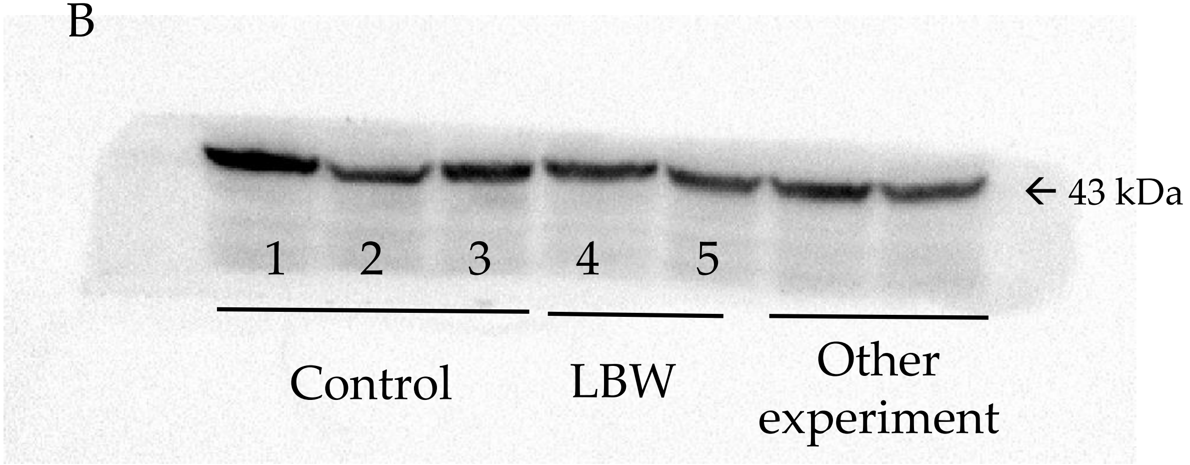

Figure S2. Immunoblot protein expression of Nrf2 (A) and corresponding β-actin (B) in liver. Control, n = 3; LBW, n = 2. The density ratio of Nrf2 to β-actin are presented as follows, band#1, 1.12; band#2, 0.49; band#3, 0.85; band#4, 0.55; band#5, 0.38.

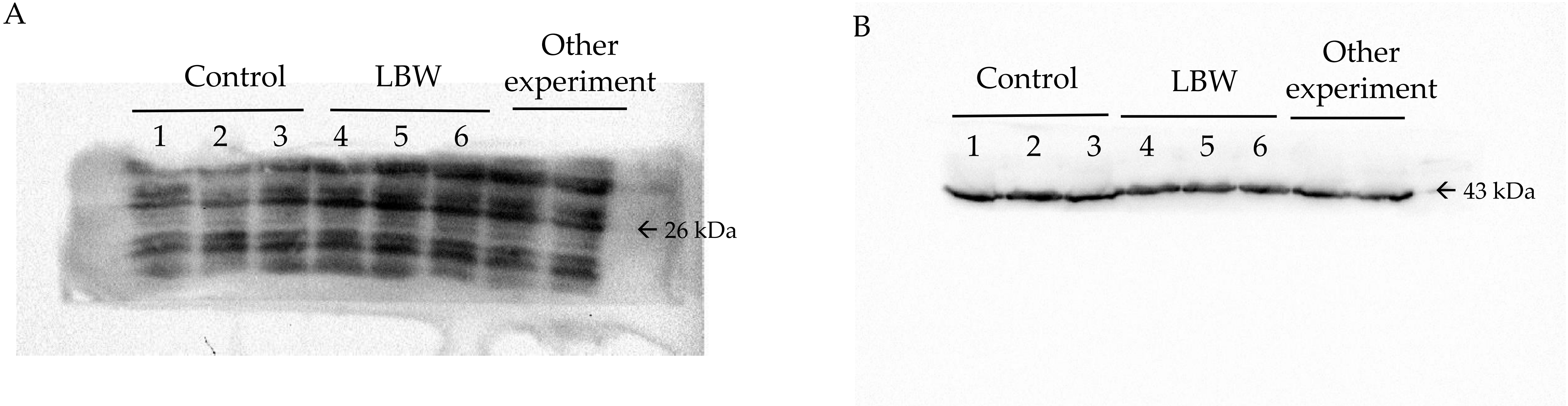

Figure S3. Immunoblot protein expression of TNFα (A) and corresponding β-actin (B) in liver. Control, n = 3; LBW, n = 3. The density ratio of TNFα to β-actin are presented as follows, band#1, 0.91; band#2, 0.75; band#3, 1.23; band#4, 1.48; band#5, 1.85; band#6, 1.57.

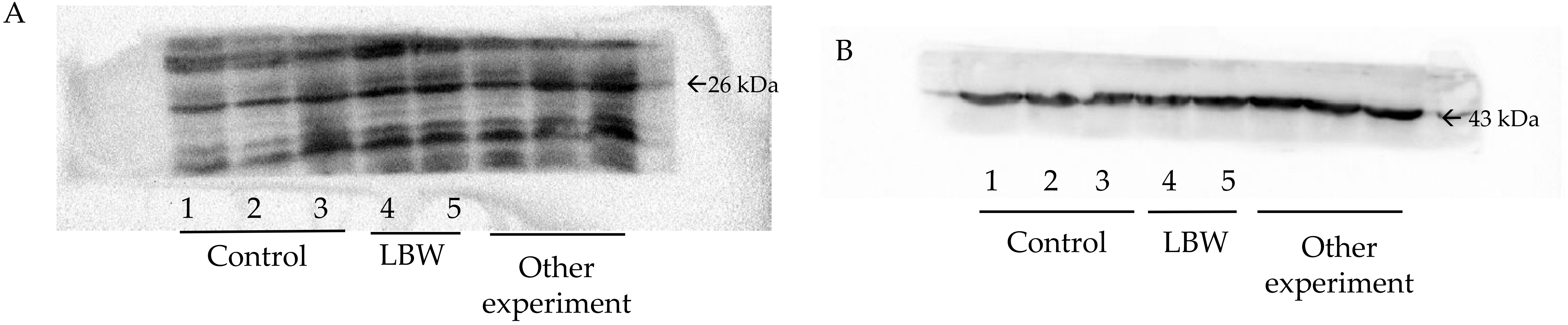

Figure S4. Immunoblot protein expression of TNFα (A) and corresponding β-actin (B) in liver. Control, n = 3; LBW, n = 2. The density ratio of TNFα to β-actin are presented as follows, band#1, 0.88; band#2, 0.96; band#3, 1.23; band#4, 1.32; band#5, 1.28.

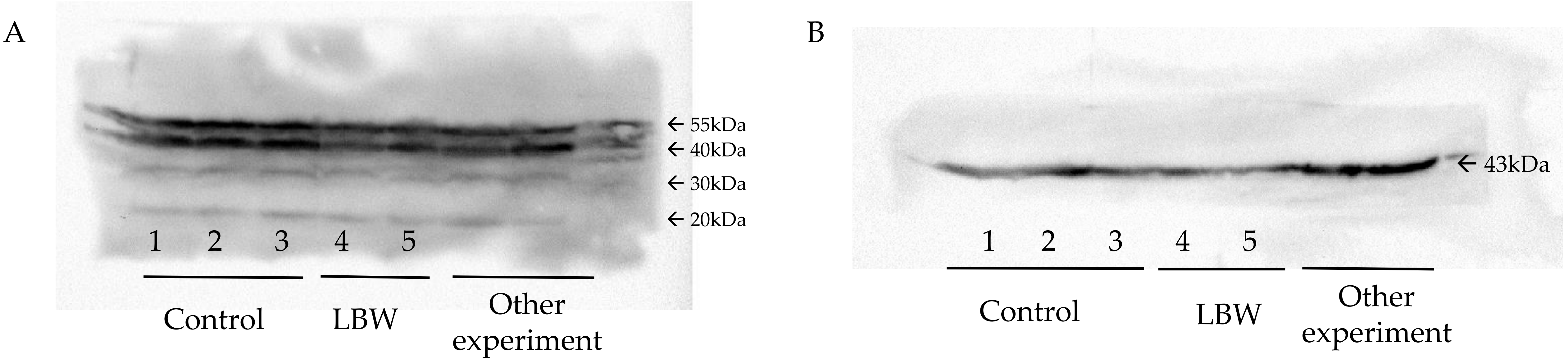

Figure S5. Immunoblot protein expression of oxidative phosphorylation complexes. A. immunoblots for the complexes I (CI, NDUFB8), II (CII, SDHB), IV (CIV, MTCO1) and V (CV, ATP5A). B. immunoblots for corresponding β-actin in liver. Control, n = 3; LBW, n = 2. The density ratio of CI are presented as follows, band#1, 1.18; band#2, 1.04; band#3, 0.95; band#4, 0.75; band#5, 0.69. The density ratio of CII are presented as follows, band#1, 1.53; band#2, 1.45; band#3, 1.08; band#4, 0.83; band#5, 0.56. The density ratio of CIV are presented as follows, band#1, 1.40; band#2, 1.10; band#3, 0.91; band#4, 0.65; band#5, 0.78. The density ratio of CV are presented as follows, band#1, 1.33; band#2, 1.26; band#3, 0.95; band#4, 0.71; band#5, 0.81.

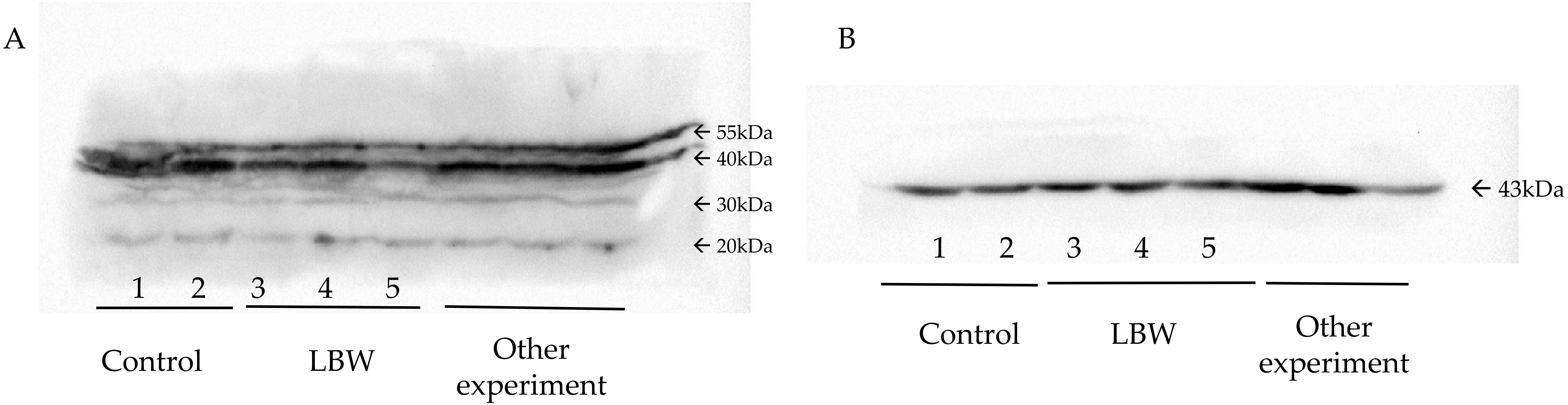

Figure S6. Immunoblot protein expression of oxidative phosphorylation complexes. A. immunoblots for the complexes I (CI, NDUFB8), II (CII, SDHB), IV (CIV, MTCO1) and V (CV, ATP5A). B. immunoblots for corresponding β-actin in liver. Control, n = 2; LBW, n = 3 The density ratio of CI are presented as follows, band#1, 0.94; band#2, 0.85; band#3, 0.99; band#4, 1.41; band#5, 0.95. The density ratio of CII are presented as follows, band#1, 1.03; band#2, 0.57; band#3, 0.42; band#4, 0.67; band#5, 0.79. The density ratio of CIV are presented as follows, band#1, 0.73; band#2, 1.01; band#3, 0.76; band#4, 0.76; band#5, 0.71. The density ratio of CV are presented as follows, band#1, 0.88; band#2, 0.68; band#3, 0.85; band#4, 0.80; band#5, 0.72.
