# Supplementary material for: Hepatic Lipid Accumulation and Dysregulation Associate with Enhanced Reactive Oxygen Species and Pro-Inflammatory Cytokine in Low-Birth-Weight Goats
Source: Animals (Basel). 2022 Mar 18;12(6):766. doi: 10.3390/ani12060766 (PMC8944635; doi:10.3390/ani12060766)
Supplement: Supplementary file 1 [file animals-12-00766-s001.zip › Table S1.pdf]

Table S1. Nucleotide sequences of primers used for real-time qPCR.

Gene	Primer Sequence	Accession No.	Product size (bp)
APOA1	F: TGAAAGCTGTGGTGCTGACCTT	XM_018059749.1	241
	R: GTTTTCCCAAAGCGGAGGCT		
APOA5	F: CCGAGAGACTGAGCAAGTCC	XM_018058993.1	128
	R: CTGGACTATGGTCGCGAAGG		
FABP3	F: GTCGGTTTTGCTACCAGGCA	NM_001285701.1	152
	R: GCCGTGGTCTCATCGAACTC		
GPx2	F: TGCAATGCCGCTTCCCCAGG	XM_005685982.3	115
	R: GGGCGGACGTACTTGAGGCTG		
GPx3	F: GTCGAAGACGGACTGCCACGC	XM_005683183.3	153
	R: TGGCCCGTCAGGCCTCAGTAG		
HO-1	F: ACACCCAGGCGGAGAATG	NM_001285567.1	126
	R: CTCCTGGAGTCGCTGAACATAG		
LCN2	F: AGGAAGACGGCAGCTACAAC	XM_018055847.1	131
	R: CTTCAGGGGTCAGCTCCTTG		
Nrf2	F: TGACAATGAGGTTTCTTCG	XM_013968675.2	129
	R: GTGGCTACCTGAACGAACA		
PLIN5	F: TGGAAGAGCAGCGGAAACAT	XM_018050878.1	116
	R: CTGCCTCAGTTTCCCCAGAG		
PPP1R14D	F: TCTTCAAGCCCTGCTTCCTG	XM_018054436.1	108
	R: CTCGGAGTCCGTGGATGATG		
SOD2	F: ACAGCATCTTCTGGACAAATC	XM_018053428.1	131
	R: ACCAACAGATACAGCAGTCAG		
S15	F: TGGTTGGCGTCTACAACGG	XM_018050438.1	242
	R: CTTCACGGGCTTGTAGGTGAT		
mtDNA	F: CAAAGCCACTCTCACCCGAT	AB044308.1	250
	R: GTCTGGGTCTCCGAGTAGGT		
nDNA	F: GCACGTAGTCTGTGACTGCT	NC030818.1	150
	R: CCGAAATGCCGTCTTTAGCG		

APOA, apolipoprotein; FABP3, fatty acid binding protein 3; GPx, glutathione peroxidase; HO-1, heme oxygenase 1; LCN2, lipocalin 2; Nrf2, Nuclear factor erythroid 2-related factor 2; PLIN5, perilipin 5; PPP1R14D, protein phosphatase 1 regulatory inhibitor subunit 14D; SOD2, superoxide dismutase 2.
