# Supplementary material for: Hepatic Lipid Accumulation and Dysregulation Associate with Enhanced Reactive Oxygen Species and Pro-Inflammatory Cytokine in Low-Birth-Weight Goats
Source: Animals (Basel). 2022 Mar 18;12(6):766. doi: 10.3390/ani12060766 (PMC8944635; doi:10.3390/ani12060766)
Supplement: Supplementary file 1 [file animals-12-00766-s001.zip › Table S3.pdf]

Table S3. Statistics of sequencing data output.

#SampleID	ReadSum	BaseSum	GC(%)	N(%)	Q20(%)	Q30(%)
Control_1	72,632,888	21,416,679,234	49.69	0	98.10	94.31
Control_2	60,852,009	18,043,815,904	50.19	0	98.05	94.32
Control_3	66,097,330	19,558,982,796	50.35	0	97.89	93.92
LBW_1	60,898,443	17,916,116,284	49.84	0	97.87	93.84
LBW_2	61,654,853	18,208,747,750	50.04	0	97.95	94.00
LBW_3	65,508,960	19,434,035,598	48.43	0	97.83	93.71

Annotation:
The Quality Score is an overall mapping of the probability of base calling errors. The base quality value Q formula is: $Q\text{–score} = -10*\log_{10}P$, where P is the probability of base calling error. The following table shows the correspondence between the base quality value and the probability of a base call error:

Base quality score	Probability of base calling errors	Base calling accuracy
10	10%	90%
20	1%	99%
30	0.1%	99.9%
40	0.01%	99.99%
